# Zinc Finger CCCH-Type Antiviral Protein 1 Restricts the Viral Replication by Positively Regulating Type I Interferon Response

**DOI:** 10.3389/fmicb.2020.01912

**Published:** 2020-08-14

**Authors:** Baoge Zhang, Mohsan Ullah Goraya, Na Chen, Lifeng Xu, Yan Hong, Meiyi Zhu, Ji-Long Chen

**Affiliations:** ^1^Key Laboratory of Fujian-Taiwan Animal Pathogen Biology, College of Animal Sciences, Fujian Agriculture and Forestry University, Fuzhou, China; ^2^State Key Laboratory of Food Safety Technology for Meat Products, Xiamen, China; ^3^CAS Key Laboratory of Pathogenic Microbiology and Immunology, Institute of Microbiology, Chinese Academy of Sciences, Beijing, China

**Keywords:** ZC3HAV1, interferon, innate immunity, influenza virus, host factor

## Abstract

Zinc finger CCCH-type antiviral protein 1 (ZC3HAV1) is a host antiviral factor that can repress translation and promote degradation of specific viral mRNAs. In this study, we found that expression of ZC3HAV1 was significantly induced by infection with influenza A virus (IAV) and Sendai virus (Sev). It was shown that deficiency of IFNAR resulted in a dramatic decrease in the virus-induced expression of ZC3HAV1. Furthermore, transfection with poly(I:C) and treatment with interferon β (IFN-β) induced the ZC3HAV1 expression. Interference with the endogenous expression of ZC3HAV1 enhanced the replication of influenza virus by impairing the production of IFN-β and MxA, following the infection of influenza virus. In contrast, ectopic expression of ZC3HAV1 significantly restricted the replication of influenza virus by increasing the IFN-β expression. In addition, ZC3HAV1 also promoted the induction of tumor necrosis factor and interleukin 6. These results suggest that ZC3HAV1 is induced by IFN-β/IFNAR signaling during IAV and Sev infection and involved in positive regulation of IFN-dependent innate antiviral response.

## Introduction

In response to viral infection, the host innate immune system is rapidly induced to restrict the viral replication and spread. Innate immune responses are initiated by the recognition of non-self-ligands expressed by pathogens, called pathogen-associated molecular patterns (PAMPs), through pattern recognition receptors (PRRs) expressed on host cells. PRRs signaling leads to activation of interferon regulatory factors (IRFs), nuclear factor κB (NF-κB), and other cofactors that trigger the downstream regulators of innate immune responses (Goraya et al., 2015; Nguyen et al., 2017). For example, Toll-like receptors 3 and 7 (TLR3/7) in endosome recognize the foreign nucleic acid and proteins internalized via endocytosis from the extracellular space. And cytosolic retinoic acid-inducible gene (RIG) like receptors (RLRs) and NOD like-receptors detect PAMPs in the infected cells. Viral RNA and DNA are major PAMPs sensed by the PRRs that trigger the innate immunity against viral infection. Viral RNA can be recognized by endosomal TLRs and cytosolic RIG-I-like receptors (Fensterl et al., 2015). Stimulation of PRRs can activate the transcription factors NF-κB and IRF3 and IRF7 to induce the expression of interferon (IFN) and proinflammatory cytokines including tumor necrosis factor (TNF), interleukin 6 (IL-6), IL-12, and IL-1β, respectively. The interaction of IFNs with their receptors on the cell membrane initiates the Janus protein tyrosine kinase/signal transducers and activators of transcription (JAK/STAT) signaling cascade to regulate transcription of IFN-stimulated genes (ISGs) that generate an antiviral state within the cell. IFN-activated JAK/STAT signaling can also create an antiviral state in adjacent cells (Chen et al., 2018; Gargan et al., 2018).

Recently, several new antiviral ISGs have been discovered, including APOBEC3, TRIM5, ZMPSTE24, tetherin, and zinc finger CCCH-type antiviral protein 1 [ZC3HAV1, also known as zinc finger antiviral protein (ZAP)] (Gao et al., 2002; Stremlau et al., 2004; Cullen, 2006; Neil et al., 2008; Li et al., 2016). ZC3HAV1 was originally identified from a rat complementary DNA library due to its resistance to the genetically marked murine leukemia viruses (MLVs). ZC3HAV1 is encoded by ZC3HAV1, an ISG present on the human chromosome 7 (7q34), and can inhibit the replication of alphaviruses, filoviruses, hepatitis B virus, and retroviruses (Bick et al., 2003; Müller et al., 2007; Zhu et al., 2011). ZC3HAV1 directly binds to the viral mRNA and degrades it by recruiting 3′ to 5′ exoribonuclease exosome complex and PARN to shorten the poly(A) tail, resulting in viral mRNA degradation (Guo et al., 2007; Ghimire et al., 2018). Moreover, ZC3HAV1 removes the cap structure of the target mRNA by recruiting the decapping complex. Consequently, removal of the cap structure exposes the RNA body to the 5′–3′ exoribonuclease Xrn1 for degradation.

ZC3HAV1 encodes two isoforms, ZAPS and ZAPL, arising from alternative splicing, but they differ only at the C-terminus (Kerns et al., 2008; Chiu et al., 2018). In the N-terminal domain of ZC3HAV1, there are four CCCH-type zinc-finger motifs, which can bind directly to the viral RNA. ZAPL is more active against alphaviruses such as Sindbis virus (SINV) and Semliki forest virus (SFV) compared to ZAPS and has signatures of positive selection (Kerns et al., 2008; Charron et al., 2013). However, ZAPS is highly expressed in response to type I IFN stimulation and viral infection compared to ZAPL (Li et al., 2015). The antiviral activity of ZC3HAV1 depends on the presence of ZAP-responsive element (ZRE) in the viral mRNA. Viruses comprised a unique ZRE, such as MLVs, SINV, Ebola virus, HIV-1, and hepatitis B virus, are susceptible to ZC3HAV1 (Tang et al., 2017). Although ZC3HAV1 appeared to play a role in diverse cellular pathways (Li et al., 2015), its function remains largely unknown. It has been proposed that ZC3HAV1 interacts with multiple host factors such as eIF4A, eIF4G, and TRIM25 and acts as a cofactor to enhance host antiviral activity (Li et al., 2017).

Influenza A virus (IAV) can cause highly contagious respiratory disease in humans and animals, and its pandemics could have high mortality rates (Wu et al., 2015; Jakubcova et al., 2016). IAV is a member of the Orthomyxoviridae family that postures a significant threat of zoonotic infection, host switch, and the generation of pandemic viruses. IAVs can infect humans and a variety of animals, such as pigs, equines, canines, marine mammals, and birds. The evolution of IAV through self-mutation and reassortment mechanisms causes general annual epidemics and infrequent pandemics (Shao et al., 2017). In addition, IAV genome is composed of eight single-strand negative-sense RNA segments, which typically encode 17 viral proteins (Ruszkowska et al., 2016). IAV infection activates the RIG-I signaling pathway to trigger the expression of type I IFN and expression of downstream ISGs such as MxA, 2′–5′ oligoadenylate synthetase, and protein kinase R (Schneider et al., 2014). Replication of IAV is highly sensitive to the antiviral action of IFNs, which is mediated by ISGs. A previous study reported that ZC3HAV1 caused the degradation of the target viral mRNAs to inhibit replication of specific viruses (Guo et al., 2004). In this study, we found that the ZC3HAV1 acts as an antiviral ISG in response to IAV infection. The expression of ZC3HAV1 was significantly up-regulated during IAV infection, indicating that ZC3HAV1 is an influenza virus-inducible factor. Furthermore, the ectopic expression of ZC3HAV1 restricted the replication of IAV by increasing the induction of IRF3-dependent IFN-β expression. In addition, we also observed that expression of ZC3HAV1 was significantly induced by infection with Sev, and increased ZC3HAV1 inhibited the virus replication. These results suggest that antiviral role of ZC3HAV1 may be associated with positive regulation of the expression of type I IFNs and some critical ISGs.

## Materials and Methods

### Antibodies and Reagents

The primary antibodies used in this study include anti-ZC3HAV1 (ProteinTech, #16820-1-AP), anti-IRF3 (Affinity Biosciences, Cincinnati, OH, United States), anti-p-IRF3 (Cell Signaling Technology, United States), and anti-β-actin (Transgen Biotechnology, HC201). A monoclonal antibody against the NP protein of WSN influenza virus was prepared in our laboratory as described previously (Liu et al., 2019). IFN-β was obtained from Sinobiologicals (Beijing), and Vigofact was obtained from Vigorous Labs (Vigorous Biotechnology Beijing, Co., Ltd.).

### Cell Culture and Virus Infection

The 293T, A549, and MDCK cells (American Type Culture Collection, Manassas, VA, United States) were maintained in Dulbecco modified Eagle medium (DMEM) containing 10% fetal calf serum and supplemented with penicillin (100 U/mL) and streptomycin (100 mg/mL). When the cells grew to a density of approximately 80 to 90% confluence, the cells were infected with A/WSN/33 virus (WSN) at the indicated multiplicity of infection (MOI) with gentle agitation every 15 min. After adsorption for 1 h at 37°C, the cells were washed with phosphate-buffered saline (PBS) and then cultured in DMEM containing 2 μg/mL trypsin.

### Differential Expression of Selected Genes in Response to IAV

Total RNAs were extracted by TRIzol reagent (Invitrogen) from the A549 cells infected with or without influenza WSN virus for 12 h in three independent experiments, and the RNA samples were sent to Shanghai OE-Biotech, Ltd. for microarray analysis. According to the microarray data, cDNA libraries were constructed to confirm the expression levels of the selected genes. Specific primers for reverse transcriptase-polymerase chain reaction (RT-PCR) and quantitative RT-PCR (qRT-PCR) were designed using Primer 5 software (Table 1). The heat map was generated from the data of differential gene expression analysis.

**TABLE 1:** List of primers used in this study.

Primer name	Primer sequence (5′–3′)
WSN-NP F	TCAAACGTGGGATCAATG
WSN-NP R	GTGCAGACCGTGCTAAAA
Sev-NP F	ATAAGTCGGGAGGAGGTGCT
Sev-NP R	GTTGACCCTGGAAGAGTGGG
Human ZC3HAV1 F	ATCCACCTCTGTTCTGTAG
Human ZC3HAV1 R	TCTTCTCCATACTGAATCCAT
Human IRF3 F	AAGGACCCTCACGACCCACA
Human IRF3 R	TCAGAAGTACTGCCTCCACCAT
Human IFN-β F	ATTGCTCTCCTGTTGTGCTT
Human IFN-β R	GTCTCATTCCAGCCAGTGCTC
Human MxA F	CTCCGACACGAGTTCCACAA
Human MxA R	GGCTCTTCCAGTGCCTTGAT
Human TNF F	CACCACGCTCTTCTGCCTGCT
Human TNF R	TTATCTCTCAGCTCCACGCCAT
Human IL-6 F	ACAAATTCGGTACATCCTCGAC
Human IL-6 R	TGGCTTGTTCCTCACTACTCT
Human HERC5 F	ATGACTGTGGACGCTTCAGA
Human HERC5 R	CCTCAATTGCTGCCGACCTA
Human GBP1F	AGCCCTACAACTTCGGAACAG
Human GBP1R	TCTGGATTCGCCATCAGTCG
Human BATF2 F	GGGAATTTGCAGCACGAGTC
Human BATF2 R	GAGCAGGAGGCACAATCCAT
Human RSAD2 F	CAAAGAAGGTGTGTCCTGCTC
Human RSAD2 R	AGAGGTTGCCTGAACACACTC
Human ATP6V0A4 F	TCCATGTATCTCAGCACGCC
Human ATP6V0A4 R	AATCAGAAGCATCCACGGCA
Human actin-F	CTGTACGCCAACACAGTGCT
Human actin-R	GCTCAGGAGGAGCAATGATC
Mouse ZC3HAV1-F	CAGGTACATCTACCCATAACGGC
Mouse ZC3HAV1-R	GTACAGGAGTAGTGGTCTCCC
Mouse β-actin-F	CGGTTCCGATGCCCTGAGGCTCTT
Mouse β-actin-R	CGTCACACTTCATGATGGAATTGA
pNL-ZAPS F	TTTAGTGAACCGTCAGATCCGCTAGC ATGGACTACAAAGACGATGACGACAA GGCGGACCCGGAGGTGTGCTGCTT
pNL-ZAPS R	TTGTAATCCAGAGGTTGATTCTCGAGT TACTCTGGCCCTCTCTTCATCT
pNL-ZAPL F	TTTAGTGAACCGTCAGATCCGCTAGCAT GGACTACAAAGACGATGACGACAAGGC GGACCCGGAGGTGTGCTGCTT
pNL-ZAPL R	TTGTAATCCAGAGGTTGATTCTCGAGCTA ACTAATCACGCAGGCTTTGTC

### sh-RNA-Based ZC3HAV1 Knockdown A549 Cell Line

To interfere with the endogenous expression of ZC3HAV1, the short hairpin RNAs (sh-RNAs) were designed, and the sequences of the sh-RNAs specifically targeting ZC3HAV1 (NCBI accession no. BC033105) were 5′-GCACTTACCTTGCTTCCAATT, 3′-AATTGGAAGCAAGGTAAGTGC, and sh-RNAs specifically targeting luciferase were used as a control. Then sh-RNA-based knockdown cell lines were generated by infecting A549 cells with lentiviruses expressing specific sh-RNAs in pSIH-H1-GFP vector as described previously (Wang et al., 2012; Chen et al., 2016). RT-PCR, qRT-PCR, and Western blotting were performed to determine the knockdown efficiency.

### siRNA-Based Knockdown of ZC3HAV1 Isoforms

To address the role of two isoforms of ZC3HAV1, ZAPL and ZAPS, in IAV infection, specific siRNAs were synthesized (Fuzhou Sunya Biotechnology, Co., Ltd.); 50 nM of si-ZAPL (5′-AAAUUUAUCCAGGAGCUCUGAGUUC-3′) or 50 nM of si-ZAPS (5′-GAUUCUUUAUCUGAUGUCATT-3′) was transfected into A549 cells for 36 h according to the manufacturer’s instructions, and the samples were collected for further analysis.

### Plasmids Construction and Gene Overexpression

The cDNAs encoding human ZAPL and ZAPS were subcloned into the pNL-CMV vector with a Flag tag at the N-terminus to generate pNL-Flag-ZAPL and pNL-Flag-ZAPS. A549 cells were seeded into six-well plates, and transient transfection of pNL-Flag-ZAPL and pNL-Flag-ZAPS plasmids was performed by using Lipofectamine 2000 according to the manufacturer’s instruction. Protein and total RNA were, respectively, extracted from the cells infected with or without influenza WSN virus for 6 and 12 h. The ZC3HAV1 overexpression in A549 cells was analyzed by qRT-PCR or Western blotting using anti-ZC3HAV1 monoclonal antibody (MAb) (dilution of 1:2,000).

### Plaque Assay

Plaque assay was performed to quantify the viral replication in A549 cells as described previously (Qidong et al., 2019). Briefly, supernatants from A549 cells infected with influenza A/WSN/33 (WSN) virus were collected. MDCK cells were seeded in 12-well plates, and MDCK cells were then infected with the supernatants of virus-infected A549 cells for 1 h. After infection, MDCK cells were washed with PBS and grown in α-minimal essential medium containing 1.5% low-melting-point agarose (Promega, Madison, WI, United States) and TPCK (tolylsulfonyl phenylalanyl chloromethyl ketone) treated trypsin (Sigma-Aldrich, St. Louis, MO, United States). After incubation for 72 h, the numbers of plaques were counted (Wang et al., 2014).

### RT-PCR and qRT-PCR

Total RNA was isolated from A549 cells, and lungs of the mice infected with WSN virus by Trizol method (TransGen Biotech, Beijing, Co., Ltd.) according to the manufacturer’s instructions. Isolated RNA (4 μg) was reverse-transcribed into cDNA libraries by using M-MLV RT (Promega, United States) according to the manufacturer’s instructions with slight modifications. The cDNA was used for expression analysis by qRT-PCR using TransStart Green qPCR SuperMix 2X (TransGen Biotech) and PCR using Taq DNA polymerase (Takara Bio) as previously described. Human β-actin was used as references for internal standardization. The amplified products by PCR were resolved by electrophoresis on a 1% agarose gel, and when necessary, the intensity of bands was analyzed using Quantity One software (Bio-Rad, United States) as previously described (Chen et al., 2015). The primers specific for WSN-NP, ZC3HAV1, IRF3, IFN-β, MxA, IL-6, TNF, and β-actin were designed using the Primer 5 software (Table 1). The data of qRT-PCR analysis were shown as normalized ratios, which was autocalculated by LightCycler system (Roche, Switzerland) using ΔΔCT method.

### Western Blotting

Cell lysates were prepared to collect the total protein, and Western blotting was performed as previously described (Danial et al., 1995). Briefly, the protein samples were separated by 12% sodium dodecyl sulfate-polyacrylamide gel electrophoresis and then transferred onto nitrocellulose membranes. The membranes were probed with the antibodies at the appropriate dilution.

### Quantification and Statistical Analysis

All statistical analyses were performed using Microsoft Excel and R software. Student’s *t*-tests were used to determine the *p*-values. *p* < 0.05 was considered statistically significant (^∗^*p* < 0.05, ^∗∗^*p* < 0.01). The results were shown as mean ± standard error.

### Ethics Statement

The animal protocol used in this study was approved by the Research Ethics Committee of College of Animal Science, Fujian Agriculture and Forestry University (permit no. PZCASFAFU2014002). The procedures carried out in accordance with the approved guidelines.

## Results

### Expression of ZC3HAV1 Is Significantly Induced During the IAV Infection *in vitro* and *in vivo*

**FIGURE 1:**
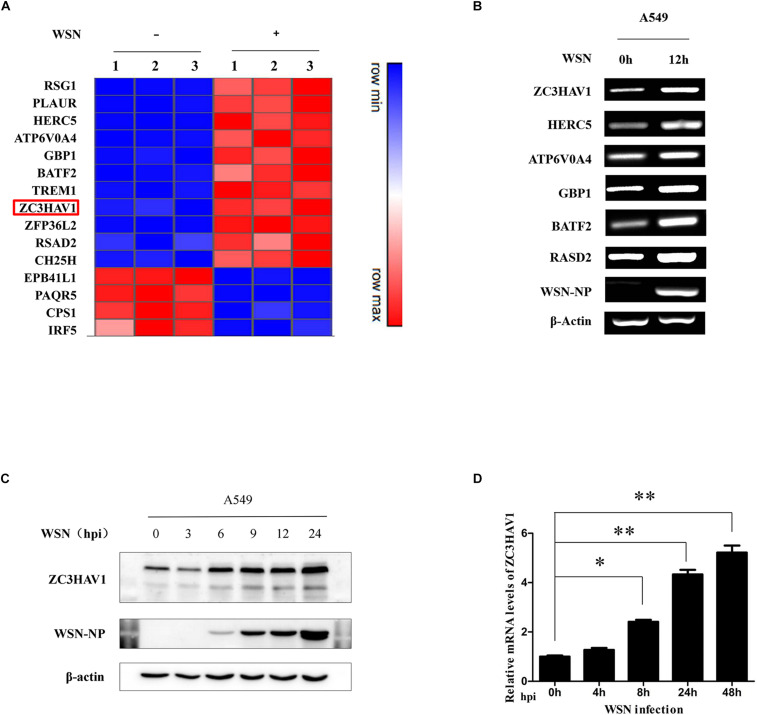
Identification of differentially expressed genes in response to influenza A/WSN/33 virus infection. **(A)** Heat map showing microarray analysis of gene expression profile in A549 cells infected with mock and influenza A/WSN/33 virus for 12 h (MOI = 1). The identified genes were differentially expressed by ≥ 2-fold (*n* = 3) between mock and virus infection. Samples were obtained from three independent experiments. **(B)** The expression of the selected genes from microarray analysis was confirmed by RT-PCR. **(C)** The expression level of ZC3HAV1 in influenza A/WSN/33 virus-infected A549 cells was measured by Western blotting. **(D)** The expression of ZC3HAV1 in influenza A/WSN/33 virus-infected BALB/c mice was measured by qRT-PCR. Data are represented as mean ± SD. ^∗^*p* < 0.05; ^∗∗^*p* < 0.01.

To identify the genes involved in the innate immune response to IAV infection, microarray analysis was performed for examining the alterations of gene expression in A549 cells infected with or without WSN virus. Compared to the control group, we identified specific immune response-related genes, which were differentially expressed with cutoff *p* ≤ 0.05 and log2 fold change ≥ 1 in WSN virus-infected A549 cells. The heat map displayed the expression levels of the selected genes (Figure 1A). The results of the microarray analysis were confirmed by using RT-PCR for examining selected genes including ZC3HAV1, HERC5, ATP6V0A4, GBP1, BATF2, and RASD2 (Figure 1B). The changes of the selected genes expression analyzed by RT-PCR were consistent with that by microarray analysis. The expression of these genes was increased after the IAV infection of A549 cells for 12 h. Since previous studies have shown that ZC3HAV1 was involved in antiviral response, we chose it for in-depth research. Thus, we infected A549 cells with WSN virus and analyzed the protein expression of ZC3HAV1 in the cells by Western blotting. Indeed, ZC3HAV1 protein level was consistently increased after the viral infection (Figure 1C). Furthermore, *in vivo* experiments were performed to determine the expression of ZC3HAV1 in mice. Similarly, we observed that ZC3HAV1 was upregulated upon the IAV infection as analyzed by qRT-PCR (Figure 1D).

### Expression of ZC3HAV1 Is Regulated by IFN-β/IFNAR Signaling

**FIGURE 2:**
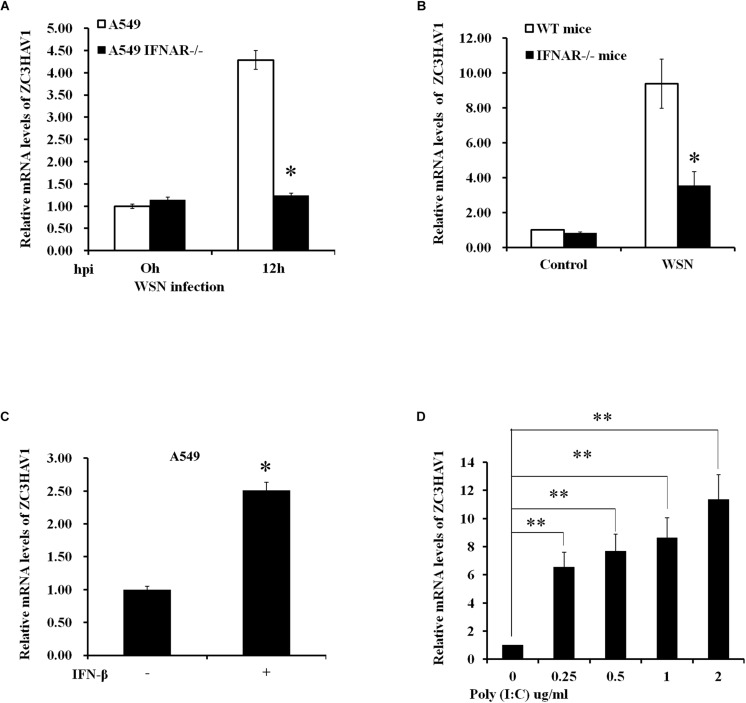
Expression of ZC3HAV1 is dependent on type I IFN signaling. **(A)** Expression level of ZC3HAV1 in WSN-infected A549 and IFNAR^–/–^ A549 cells was measured by qRT-PCR. The data were expressed as fold change relative to the level in A549 cells at 0 h post-infection. Data are represented as mean ± SD. ^∗^*p* < 0.05. **(B)** Expression level of ZC3HAV1 in wild-type and interferon A receptor knockout (IFNAR^–/–^) mice infected with or without WSN virus for 48 h was measured by qRT-PCR. Data are represented as mean ± SD. ^∗^*p* < 0.05. **(C)** Expression level of ZC3HAV1 in A549 cells treated with IFN-β was measured by qRT-PCR. Data are represented as mean ± SD. ^∗^*p* < 0.05. **(D)** ZC3HAV1 expression in A549 cells treated with poly(I:C) was examined by qRT-PCR. Data are represented as mean ± SD. ^∗∗^*p* < 0.01.

To investigate whether the expression of ZC3HAV1 is dependent of IFN-β/IFNAR signaling, we used IFNAR knockout A549 cells and IFTNAR knockout mice. The IFNAR knockout A549 cells were infected with WSN for 12 h (MOI = 1), and total RNA was collected from the infected cells. Mice were infected with WSN for 48 h, and then the lung tissues were collected, and total RNA was isolated. These samples were examined by qRT-PCR, and the results showed that the expression levels of ZC3HAV1 in IFNAR knockout A549 cells and in lung tissues of IFNAR knockout mice were significantly lower than those in the control group after influenza virus infection (Figures 2A,B). These experiments demonstrate that the expression of ZC3HAV1 is dependent on IFNAR signaling *in vitro* and *in vivo*. Furthermore, we observed that the expression of ZC3HAV1 was clearly upregulated in response to “IFN-β treatment (Figure 2C). Additionally, the results showed that mRNA expression level of ZC3HAV1 was significantly increased in A549 cells stimulated by poly:(I:C) compared with the control group, and the expression level of ZC3HAV1 was consistently increased with the increase of poly(I:C) concentration (Figure 2D). These data indicate that ZC3HAV1 may play a role in innate immunity against the IAV infection.

### Silencing ZC3HAV1 Enhances the IAV Replication

**FIGURE 3:**
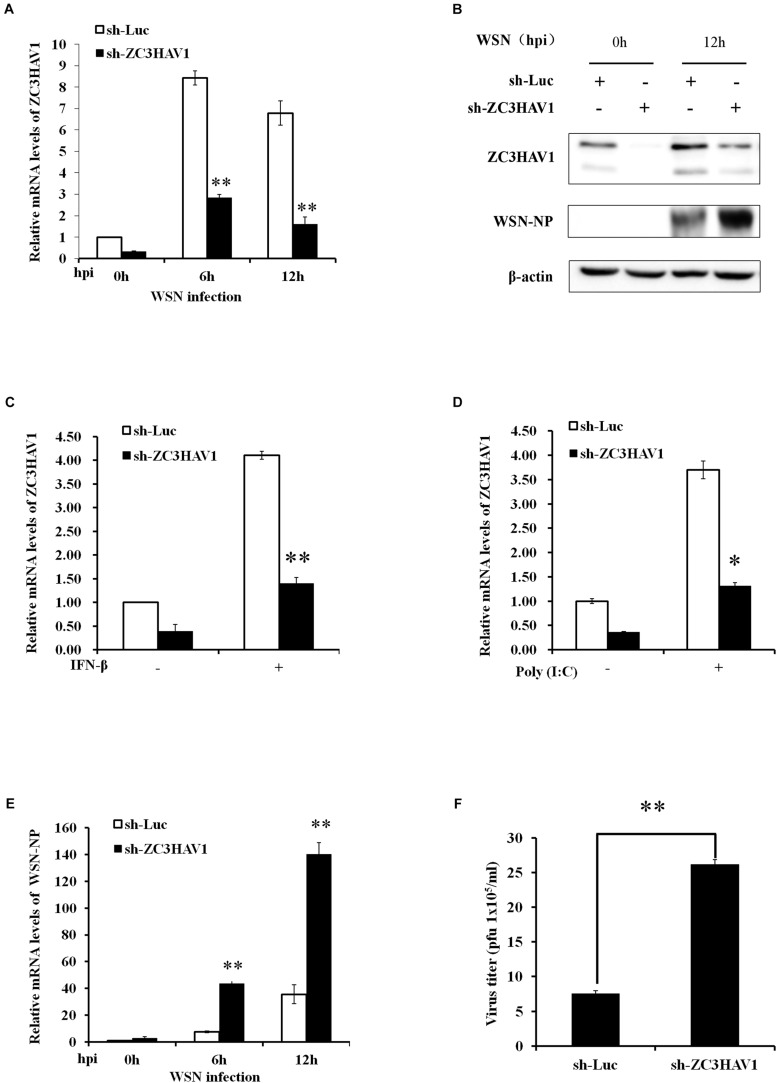
Knockdown of endogenous ZC3HAV1 expression significantly increases the replication of IAV in A549 cells. A549 cells stably expressing specific sh-RNAs targeting ZC3HAV1 or luciferase control were infected with or without WSN virus at an MOI of 1.0, and the extracts were harvested at the indicated time points after infection. **(A)** qRT-PCR was performed to measure the interference efficiency of the sh-ZC3HAV1. Data are represented as mean ± SD. ^∗∗^*p* < 0.01. **(B)** Protein extracts collected from the A549 cells expressing specific sh-ZC3HAV1 infected with WSN virus at the indicated time points (0 and 12 hpi) were subjected to Western blotting analysis with antibodies specific to ZC3HAV1 and WSN-NP. **(C,D)** qRT-PCR was performed to measure the expression of ZC3HAV1 after treatment with IFN-β and poly(I:C) in ZC3HAV1 knockdown A549 cells. Data are represented as mean ± SD. ^∗^*p* < 0.05; ^∗∗^*p* < 0.01. **(E)** A549 cells expressing specific sh-ZC3HAV1 were infected with WSN virus, and qRT-PCR was performed to detect the mRNA expression of WSN-NP. Data are represented as mean ± SD. ^∗∗^*p* < 0.01. **(F)** The cell culture supernatants derived from sh-Luc and sh-ZC3HAV1-expressing A549 cells were harvested to determine the viral titers by plaque assay using MDCK cells. Data are represented as mean ± SD. ^∗∗^*p* < 0.01.

Next, to determine the function of ZC3HAV1 in innate immunity, we generated the stable A549 cell lines expressing specific sh-RNAs targeting ZC3HAV1 or luciferase control. The positive cells were observed under fluorescent microscope to detect the expression of GFP (Supplementary Figure S1A), and qRT-PCR was performed to confirm the disruption of ZC3HAV1 expression (Supplementary Figure S1B). Then ZC3HAV1 knockdown cells were infected with WSN virus, and RNA extracts were harvested at the indicated time [0, 6, and 12 hpi (hours post infection)]. The efficiency of sh-RNA-based knockdown was further examined by qRT-PCR and Western blotting in cells after WSN virus infection (Figures 3A,B). Compared with the control sh-RNA targeting luciferase, the specific sh-ZC3HAV1 caused a significant decrease in the expression of ZC3HAV1 in cells infected with WSN virus. The ZC3HAV1 knockdown A549 cells showed a significant lower expression of ZC3HAV1 even in response to IFN-β and poly(I: C) treatment (Figures 3C,D).

Because results presented above revealed that IAV infection induced the expression of ZC3HAV1, we determined whether altering ZC3HAV1 expression had any effect on IAV replication. To this end, the ZC3HAV1 knockdown A549 cells were infected with the WSN virus, and total RNA and proteins were collected from the ZC3HAV1 knockdown and luciferase control A549 cells at the indicated time points (0, 6, and 12 hpi). Expression of IAV nuclear protein (NP) in ZC3HAV1 knockdown cells was detected using qRT-PCR. Interestingly, the results showed higher NP mRNA level in sh-ZC3HAV1 treated A549 cells than that in sh-Luc-treated cells (Figure 3E). Because NP is an indicator of the replication of IAV, increased NP mRNA level by silencing of endogenous ZC3HAV1 suggested an increase in viral replication. To confirm this observation, viral load in culture supernatants of ZC3HAV1 knockdown A549 cells was compared to supernatants of luciferase control cells. The plaque assay showed a higher number of plaque-forming units of WSN in sh-ZC3HAV1-treated A549 cells as compared to the luciferase control (Figure 3F). In addition, data obtained from Western blotting showed that NP protein expression was higher in ZC3HAV1 knockdown cells than that in the control (Figure 3B). These observations indicate that knockdown of ZC3HAV1 is in favor replication of IAV. Because ZC3HAV1 encodes ZAPS and ZAPL, two isoforms, we further investigated whether ZAPL or ZAPS had an inhibitory effect on viral NP expression. Thus, A549 cells were transiently transfected with specific siRNA-ZAPL and siRNA-ZAPS or control siRNA and challenged with WSN virus. Interestingly, we observed that it was ZAPS instead of ZAPL, which inhibited mRNA expression of viral NP (Supplementary Figure S1C).

### Influence of ZC3HAV1 Knockdown on IRF3 Signaling and Some Cytokine Expression

**FIGURE 4:**
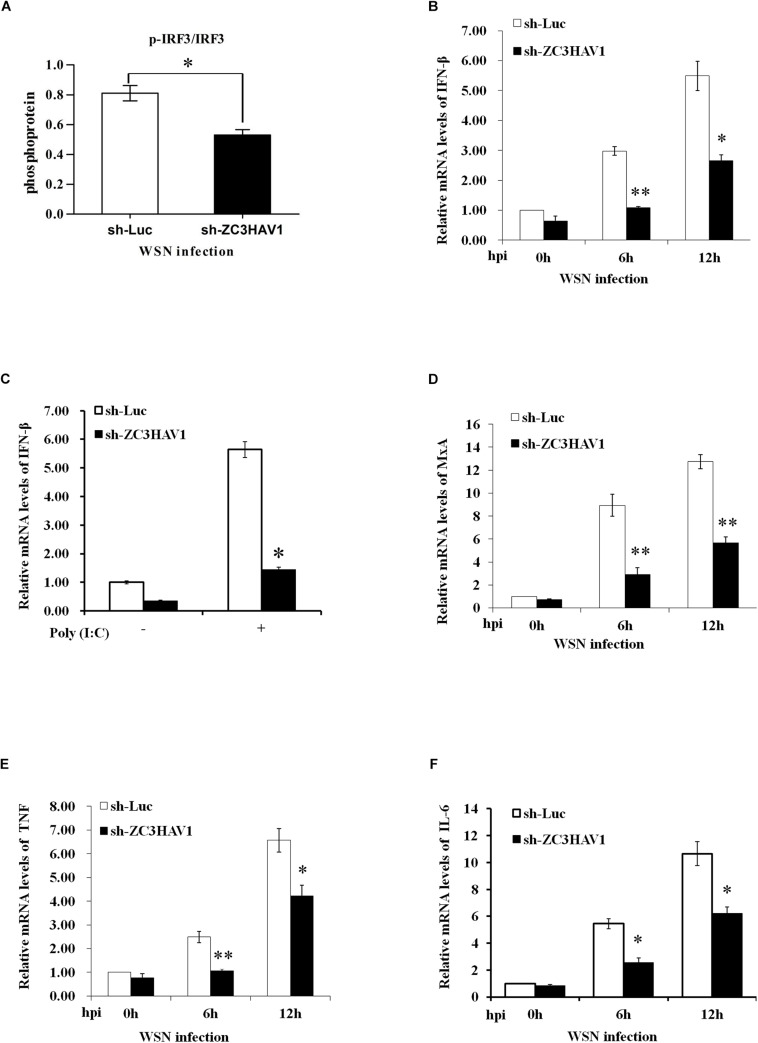
ZC3HAV1 knockdown has significant effect on IRF3 signaling and some cytokine expression in IAV-infected cells. **(A)** Bar graph shows the relative abundance of p-IRF3 protein (normalized to that of IRF3) from three experiments by using ImageJ software in A549 cells expressing sh-ZC3HAV1 or sh-Luc in response to WSN infection. **(B)** qRT-PCR was performed to detect the mRNA expression of IFN-β in A549 cells expressing sh-ZC3HAV1 or sh-Luc in response to WSN infection. Data are represented as mean ± SD. ^∗^*p* < 0.05; ^∗∗^*p* < 0.01. **(C)** The expression of IFN-β mRNA was significantly reduced after poly(I:C) stimulation in sh-ZC3HAV1-expressedA549 cells, which was assessed by qRT-PCR. Data are represented as mean ± SD. ^∗^*p* < 0.05. **(D)** qRT-PCR was performed to detect the expression of MxA mRNA in A549 cells expressing sh-ZC3HAV1 or sh-Luc in response to WSN infection. Data are represented as mean ± SD. ^∗∗^*p* < 0.01. **(E,F)** qRT-PCR analysis determined that the expression of TNF and IL-6 mRNAs was suppressed in A549 cells expressing sh-ZC3HAV1 in comparison to the cells expressing sh-Luc after the IAV infection. Data are represented as mean ± SD. ^∗^*p* < 0.05; ^∗∗^*p* < 0.01.

To explore the function of ZC3HAV1 in innate immune signaling, the total RNA were extracted from the ZC3HAV1 knockdown A549 cells and sh-Luc control cells infected with WSN virus (MOI = 1) at the indicated time points. The results show that the protein expression level of p-IRF3 was reduced in ZC3HAV1 knockdown cells after the viral infection by using ImageJ software (Figure 4A and Supplementary Figure S1D).

Interestingly, silencing endogenous ZC3HAV1 resulted in a significant increase of viral replication and caused a significant suppression of the expression of IFN-β mRNA in A549 cells at 6 and 12 h after the infection, as evidenced by data from qRT-PCR and RT-PCR compared to the luciferase control cells (Figure 4B and Supplementary Figure S1B). Knockdown of ZC3HAV1 also resulted in a significantly reduced expression of IFN-β mRNA in response to poly(I: C) stimulation, as assessed by qRT-PCR (Figure 4C). A previous study has reported that induction of MxA, a critical ISG, is dependent on the expression of type I IFN or type III IFN (Holzinger et al., 2007). Therefore, we evaluated the expression of MxA and found that its expression was significantly reduced in ZC3HAV1 knockdown cells in response to IAV infection (Figure 4D and Supplementary Figure S1B). Furthermore, we examined the expression levels of other cytokines, such as TNF and IL-6. The expression of TNF and IL-6 was also decreased in ZC3HAV1 knockdown cells as compared with that in control cells (Figures 4E,F and Supplementary Figure S1B). Moreover, we explored the influence of ZAPL and ZAPS on the expression of IFN-β, MxA, TNF, and IL-6 at 12 h after infection. We observed that silencing ZAPS but not ZAPL caused a significant decrease in expression of IFN-β and MxA (Supplementary Figures S1E,F). It appeared that knockdown of ZAPL or ZAPS had inhibitory effect on expression of TNF and IL-6 (Supplementary Figures S1G,H).

### Overexpression of ZC3HAV1 Significantly Inhibits the IAV Replication

**FIGURE 5:**
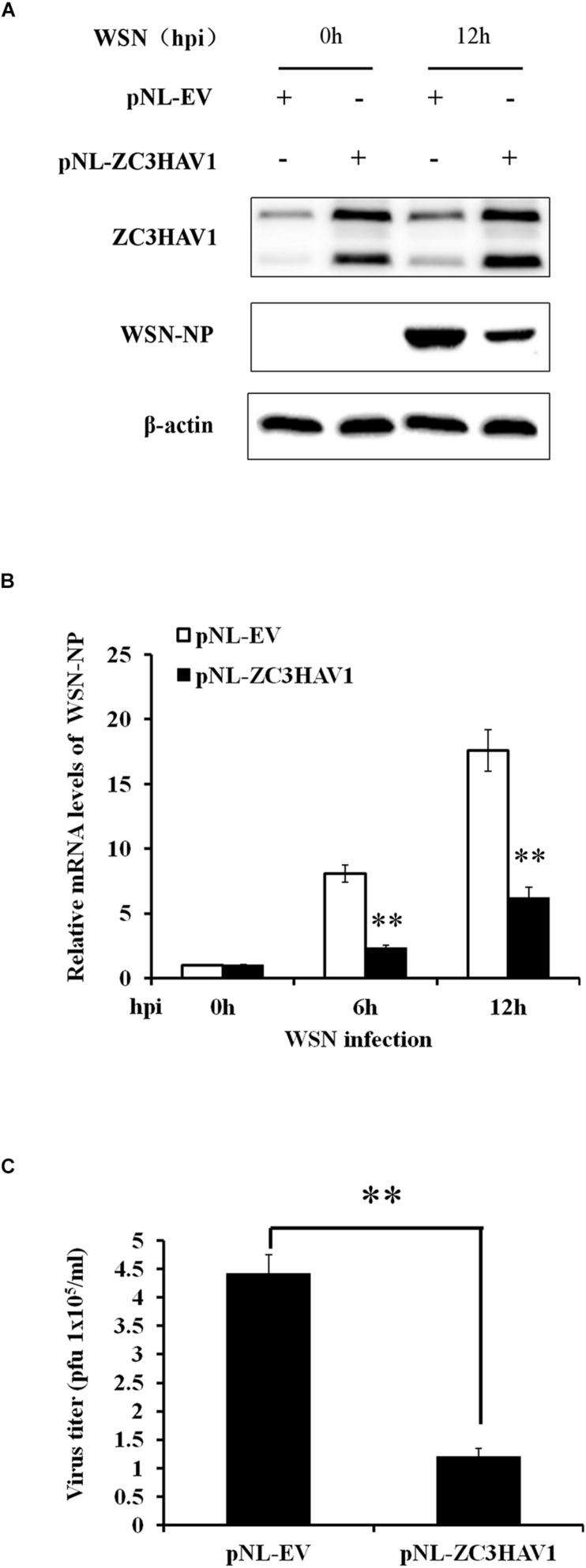
Overexpression of ZC3HAV1 restricts the replication of WSN virus. A549 cells stably expressing ZAPL, ZAPS, or empty vector were infected with WSN virus at an MOI of 1.0 and harvested at the indicated time points (0, 6, and 12 hpi). **(A)** Expression of ZC3HAV1 and WSN-NP in A549 cells was examined by Western blotting with antibodies against ZC3HAV1 and WSN-NP. **(B)** The mRNA expression of WSN-NP was examined by qRT-PCR in WSN-infected A549 cells stably expressing ZAPL, ZAPS, or empty vector. Data are represented as mean ± SD. ^∗∗^*p* < 0.01. **(C)** The viral titers in culture supernatants from WSN-infected A549 cells stably expressing ZAPL, ZAPS, or empty vector were determined by plaque assay using MDCK cells. Data are represented as mean ± SD. ^∗∗^*p* < 0.01.

Because silencing of ZC3HAV1 increased the susceptibility of A549 cells to IAV infection, we further investigated whether forced expression of ZC3HAV1 could inhibit the IAV replication. Thus, A549 cells were transiently transfected with a construct overexpressing ZAPL, ZAPS, or empty vector and challenged with WSN virus. The Western blotting and RT-PCR results showed that ZC3HAV1, which encodes ZAPL and ZAPS, successfully overexpressed in A549 cells transfected with pNL-ZAPL and pNL-ZAPS (Figure 5A and Supplementary Figure S2A). We found that the exogenous expression of ZC3HAV1 significantly reduced the expression of mRNA and protein of the viral NP, suggesting the suppression of the IAV replication (Figures 5A,B). To confirm this finding, virus titers in cell culture supernatants were determined by plaque assay. The results of plaque assay showed that the overexpression of ZC3HAV1 in A549 cells significantly restricted the replication of IAV (Figures 5A,C). Interestingly, we found that ectopic expression of ZAPS but not ZAPL significantly reduced the mRNA level of viral NP (Supplementary Figure S2B).

### Overexpression of ZC3HAV1 Enhances IRF3 Signaling and Some Cytokine Expression

**FIGURE 6:**
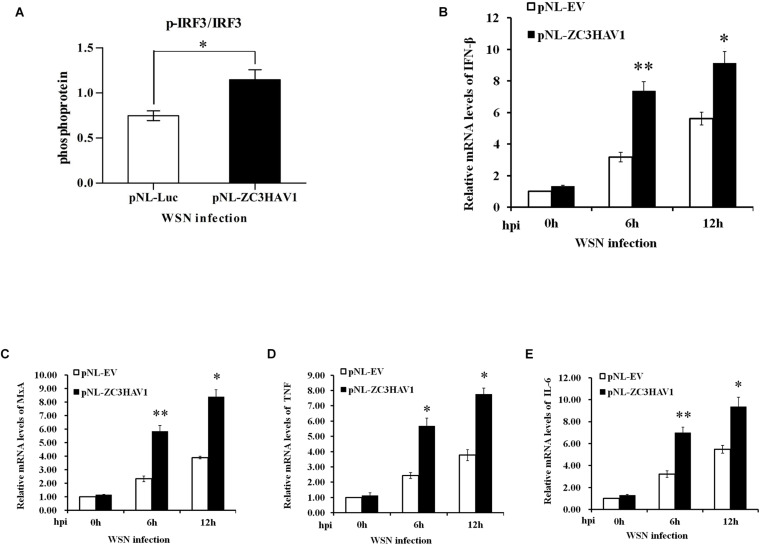
Exogenous expression of ZC3HAV1 enhances the expression of antiviral innate immunity and inflammation. **(A)** A549 cells overexpressing ZAPL, ZAPS, or empty vector were infected with WSN. Bar graph shows the relative abundance of p-IRF3 protein (normalized to that of IRF3) from three experiments by using ImageJ software in A549 cells with exogenous expression of ZAPL, ZAPS, and empty vector after the virus infection. **(B,C)** qRT-PCR was performed to detect the expression of IFN-β and MxA mRNA in A549 cells with exogenous expression of ZC3HAV1 and empty vector after the virus infection. Data are represented as mean ± SD. ^∗^*p* < 0.05; ^∗∗^*p* < 0.01. **(D,E)** qRT-PCR analysis determined that the expression of TNF and IL-6 mRNAs was enhanced in ZC3HAV1-expressing A549 cells in comparison to empty vector control. Data are represented as mean ± SD. ^∗^*p* < 0.05; ^∗∗^*p* < 0.01.

Next, we analyzed the effects of ZC3HAV1 overexpression on the expression of IRF3. We found that the protein expression level of p-IRF3 was higher in ZC3HAV1-overexpressed cells after the viral infection by using ImageJ software (Figure 6A and Supplementary Figure S2C). Additionally, we examined the effects of ZC3HAV1 on the expression of type I IFNs using RT-PCR and qRT-PCR. Overexpression of ZC3HAV1 in A549 cells markedly enhanced the WSN virus-induced expression of IFN-β mRNA (Figure 6B and Supplementary Figure S2A). Overexpression of ZC3HAV1 in A549 cells resulted in an increased induction of MxA mRNA (Figure 6C and Supplementary Figure S2A), following the infection with the IAV. We have also found that the expression levels of TNF and IL-6 were significantly increased in ZC3HAV1 overexpressed cells (Figures 6D,E and Supplementary Figure S2A). These results indicate that ZC3HAV1 is a positive regulator of IRF3-mediated innate immune response to IAV infection. Furthermore, we observed that overexpression of ZAPS rather than ZAPL significantly increased the expression of IFN-β and MxA mRNA (Supplementary Figures S2D,E). Overexpression of both ZAPL and ZAPS appeared to enhance the expression of TNF and IL-6 mRNA in A549 cells (Supplementary Figures S2F,G).

### ZC3HAV1 Can Also Be Induced by Sendai Virus and May Inhibit the Virus Replication

**FIGURE 7:**
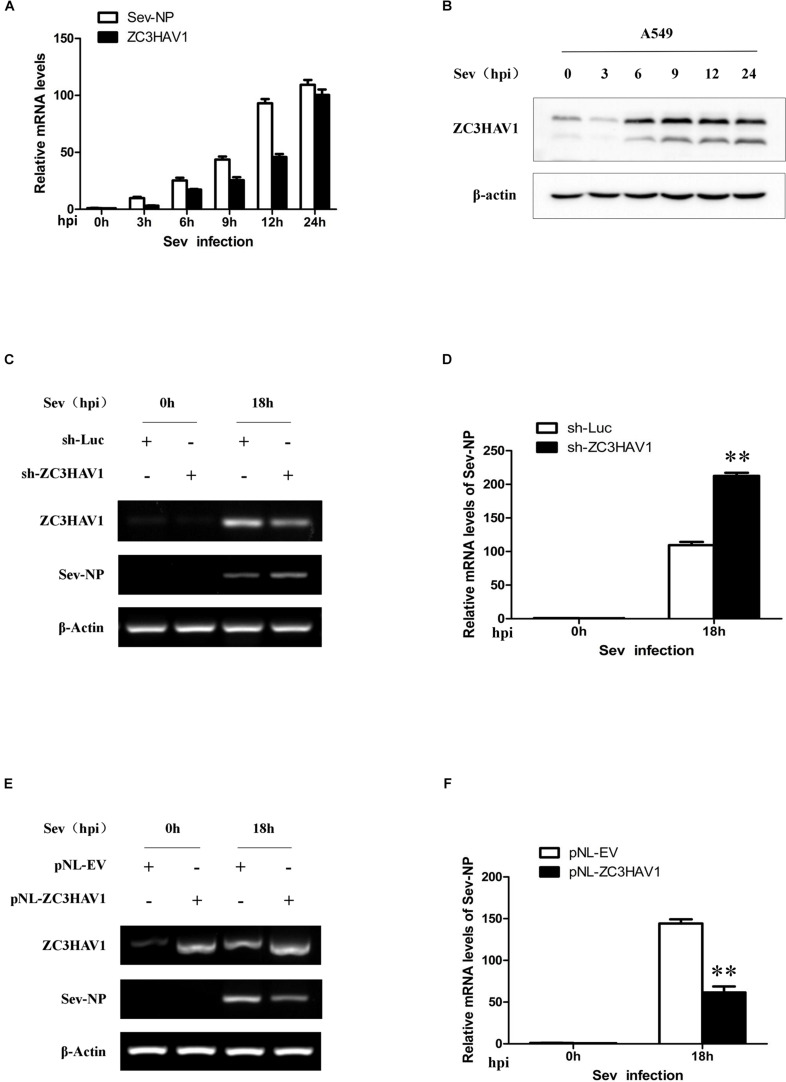
Expression of ZC3HAV1 is induced by the infection of Sev and inhibits the virus replication. **(A)** qRT-PCR was performed to detect the mRNA expression of Sev-NP and ZC3HAV1 in the virus-infected A549 cells at 0, 3, 6, 9, 12, and 24 hpi. **(B)** The expression level of ZC3HAV1 in Sev-infected A549 cells was measured by Western blotting. **(C,D)** A549 cells expressing specific sh-ZC3HAV1 were infected with Sev. RT-PCR and qRT-PCR were then performed to detect the mRNA expression of ZC3HAV1 and Sev-NP. Data are represented as mean ± SD. ^∗∗^*p* < 0.01. **(E,F)** A549 cells overexpressing ZC3HAV1 were infected with Sev, and RT-PCR and qRT-PCR were performed to detect the mRNA expression of ZC3HAV1 and Sev-NP. Data are represented as mean ± SD. ^∗∗^*p* < 0.01.

To further determine the role of ZC3HAV1 in the innate antiviral immunity, A549 cells was also infected with Sev (MOI = 1). Total RNA and protein were respectively extracted from the cells infected with or without Sev at the indicated time points (0, 3, 6, 9, 12, and 24 hpi), and the expression of ZC3HAV1 was analyzed by qRT-PCR and Western blotting. Interestingly, Sev infection also significantly induced the expression of ZC3HAV1 mRNA and protein (Figures 7A,B). Expression of ZC3HAV1 induced by Sev was upregulated in a virus infection time-dependent manner. These results suggest that ZC3HAV1 may also be involved in the antiviral innate immunity against Sev.

To further determine the role of ZC3HAV1 in antiviral innate immunity against Sev, A549 cells stably expressing specific sh-RNAs targeting ZC3HAV1 or luciferase control were infected with or without Sev, and the extracts were harvested at 0 and 18 hpi. Interference efficiency of sh-ZC3HAV1 was examined by RT-PCR (Figure 7C). Interestingly, RT-PCR and qRT-PCR results showed higher expression of Sev nucleocapsid protein in sh-ZC3HAV1 treated A549 cells than that in sh-Luc treated cells (Figures 7C,D). In contrast, RT-PCR analysis showed that expression of Sev NP was reduced in ZC3HAV1 overexpression cells as compared with the control cells (Figure 7E). Similar results were obtained from qRT-PCR analysis (Figure 7F). Nucleocapsid protein was considered as an index for the replication of Sev. These data indicate that ZC3HAV1 may also play an important role in host defense against Sev.

## Discussion

In response to viral infection, host cellular PRRs recognize PAMPs of invading viruses. Usually viral PAMPs are nucleic acids or derived from viral DNA or RNA genomes. PRRs are located in various cellular compartments and activate the transcription factors IRF3/7 and NF-κB following the recognition of invading virus (Schneider et al., 2014; Zhou et al., 2014; Sok et al., 2018). The activation of the transcription factors ultimately leads to the expression of IFNs and various ISGs, which plays a key role in restricting the replication of invading viruses. IAV infection activates these transcription factors regulated by innate immune signaling and eventually induces the expression of various IFNs and ISGs. Such innate immunity is the first line of defense against viral infection. In this study, we observed that infection with WSN virus significantly upregulated the expression of ZC3HAV1 in A549 cells, indicating that ZC3HAV1 is an influenza virus-inducible host factor. Previous studies have shown that ZC3HAV1 could repress translation and promote degradation of specific viral mRNAs and thereby suppress the viral infection (Guo et al., 2004; Zhu et al., 2012). However, the mechanism underlying interaction between virus and host is complicated. For example, various viruses have evolved to escape the host innate immunity (Goraya et al., 2015). Thus, the precise molecular mechanism by which ZC3HAV1 interacts with IAV is still not fully understood.

Here, we examined the antiviral activity of endogenous ZC3HAV1 against IAV replication by knocking down ZC3HAV1 in A549 cells. Using plaque assay, we observed that disruption of endogenous ZC3HAV1 expression enhanced replication of the IAV in A549 cells. By contrast, overexpression of the ZC3HAV1 in A549 cells showed the potent inhibitory effect on the replication of the IAV. Previously, ZC3HAV1 was known to directly bind with the RIG-I after ligand stimulation (Hayakawa et al., 2011). Assembly of RIG-I and MDA5 activates downstream IRFs and other transcription factors for the transcription of genes encoding IFN-α/β and ISGs (Johnson and Gale, 2006). In the present study, we found that the expression level of p-IRF3 protein was significantly decreased in ZC3HAV1 knockdown cells as compared to the control cells, whereas ectopic expression of ZC3HAV1 increased the expression level of p-IRF3 protein. We observed that the induction of cytokines such as TNF and IL-6 that play an important role in the inflammatory process, in response to IAV infection, was diminished in ZC3HAV1 knockdown A549 cells. The expression of TNF and IL6 can be induced by IAV infection, which contributes to host innate immunity, but also the pathogenesis of influenza virus when excessive production (Tisoncik et al., 2012; Lauder et al., 2013). Taken together, our data suggest that ZC3HAV1 may be an essential component of the IRF3-mediated innate immune response, affecting the expression of the IFN-β, MxA, TNF, and IL6.

The activation of IRFs leads to the robust expression of type I IFNs. It is well-known that type I IFN is critical for activation of antiviral responses (Sadler and Williams, 2008; Schoggins and Rice, 2011). Type I IFN stimulates various antiviral effectors, known as ISGs. Each ISG has a specific mechanism of controlling the replication of viruses (Schneider et al., 2014). In the present study, we detected that sh-RNA-mediated knockdown of ZC3HAV1 expression reduced the induction of IFN-β mRNA in response to the infection of IAV. The expression of MxA in response to influenza virus is associated with the regulation of type I IFN signaling (Holzinger et al., 2007). The induction levels of MxA, TNF, and IL-6 were also lower in ZC3HAV1 knockdown A549 cells than those in the control cells infected with the IAV. By contrast, we noticed the higher expression of viral NP mRNA in sh-ZC3HAV1-treated A549 cells than that in the control cells. On the other hand, overexpression of ZC3HAV1 in A549 cells induced higher levels of IFN-β and MxA mRNA after IAV infection. As expected, the A549 cells expressing ZC3HAV1 showed a significant suppression of the IAV replication. Similarly, the expression of ZC3HAV1 enhanced the expression of several genes, including genes encoding type I IFN, MxA, and proinflammatory cytokines IL-6, and TNF. These data reveal that ZC3HAV1 plays an important role in antiviral innate immune responses against influenza virus. In addition, we found that infection of Sev can induce the expression of ZC3HAV1 in A549 cells. Interference with ZC3HAV1 also significantly increased the replication of Sev. On the contrary, overexpression of ZC3HAV1 significantly inhibited the replication of Sev. Furthermore, we observed that both isoforms of ZC3HAV1, ZAPS and ZAPL, were induced by infections with IAV and Sev, but it appeared that ZAPS other than ZAPL was a potent regulator of IFN-β and MxA expression during the viral infection. This is consistent with the findings from previous studies (Hayakawa et al., 2011; Schwerk et al., 2019). It has been revealed that ZAPS is associated with RIG-I to promote the ATPase activity of RIG-I, leading to robust activation of IRF3 and NF-κB transcription factors that govern the expression of type I IFNs. However, the precise role of ZAPL and the precise mechanism underlying its involvement in innate immunity against IAV still remain to be determined.

Previous reports have also revealed that ZC3HAV1 can suppress other viruses such as hepatitis C virus and Japanese encephalitis virus (Kato et al., 2006; Hayakawa et al., 2011). Based on these findings, we speculate that ZC3HAV1 is likely implicated in host defense against many viruses. However, the mechanism underlying ZC3HAV1 interference of viral replication remains to be further clarified. In addition, although it has been shown that ZAPS is a key regulator of RIG-I signaling during the innate antiviral immune response, further researches are required to better understand the function of ZC3HAV1 in other PRR-mediated innate immune signaling.

## Data Availability Statement

The datasets generated for this study are available on request to the corresponding author.

## Ethics Statement

The animal study was reviewed and approved by the Research Ethics Committee of College of Animal Science, Fujian Agriculture and Forestry University (permit no. PZCASFAFU2014002).

## Author Contributions

BZ and MG conceived and designed the study. BZ, MG, and NC performed the experiments. BZ and NC analyzed the data. LX, YH, and MZ performed the statistics. MG and NC edited the manuscript. J-LC designed the study and provided the frame for the manuscript and critically revised the manuscript. All authors read and approved the final manuscript.

## Conflict of Interest

The authors declare that the research was conducted in the absence of any commercial or financial relationships that could be construed as a potential conflict of interest.

## Supplementary Material

FIGURE S1 **(A)** The positive cells were observed under fluorescent microscope to detect the expression of GFPs. **(B)** RT-PCR was performed to detect the expression of ZC3HAV1, IFN-β, MxA, TNF, and IL-6 mRNAs in A549 cells expressing sh-ZC3HAV1 or sh-Luc in response to WSN infection. **(D)** Western blotting was performed to detect the protein expression of IRF3 and p-IRF3 in ZC3HAV1 knockdown cells after the viral infection. **(C,E–H)** qRT-PCR was performed to examine the mRNA levels of viral NP, IFN-β, MxA, TNF, and IL-6 in A549 cells expressing either si-NC, si-ZAPL, or si-ZAPS after the WSN infection. Data are represented as mean ± SD. **p* < 0.05; ***p* < 0.01.

FIGURE S2 **(A)** RT-PCR was performed to detect the expression of ZC3HAV1, IFN-β, MxA, TNF, and IL-6 mRNAs in A549 cells expressing pNL-ZC3HAV1 or pNL-EV in response to WSN infection. **(C)** Western blotting was performed to detect the protein expression of IRF3 and p-IRF3 in ZC3HAV1-overexpressed cells after the viral infection. **(B,D–G)** qRT-PCR was performed to examine the mRNA expression of viral NP, IFN-β, MxA, TNF, and IL-6 in A549 cells overexpressing either pNL-EV, pNL-ZAPL, or pNL-ZAPS. Data are represented as mean ± SD. **p* < 0.05; ***p* < 0.01.
