# Supplementary figures and images for: Zinc Finger CCCH-Type Antiviral Protein 1 Restricts the Viral Replication by Positively Regulating Type I Interferon Response
Source: Front Microbiol. 2020 Aug 14;11:1912. doi: 10.3389/fmicb.2020.01912 (PMC7456897; doi:10.3389/fmicb.2020.01912)

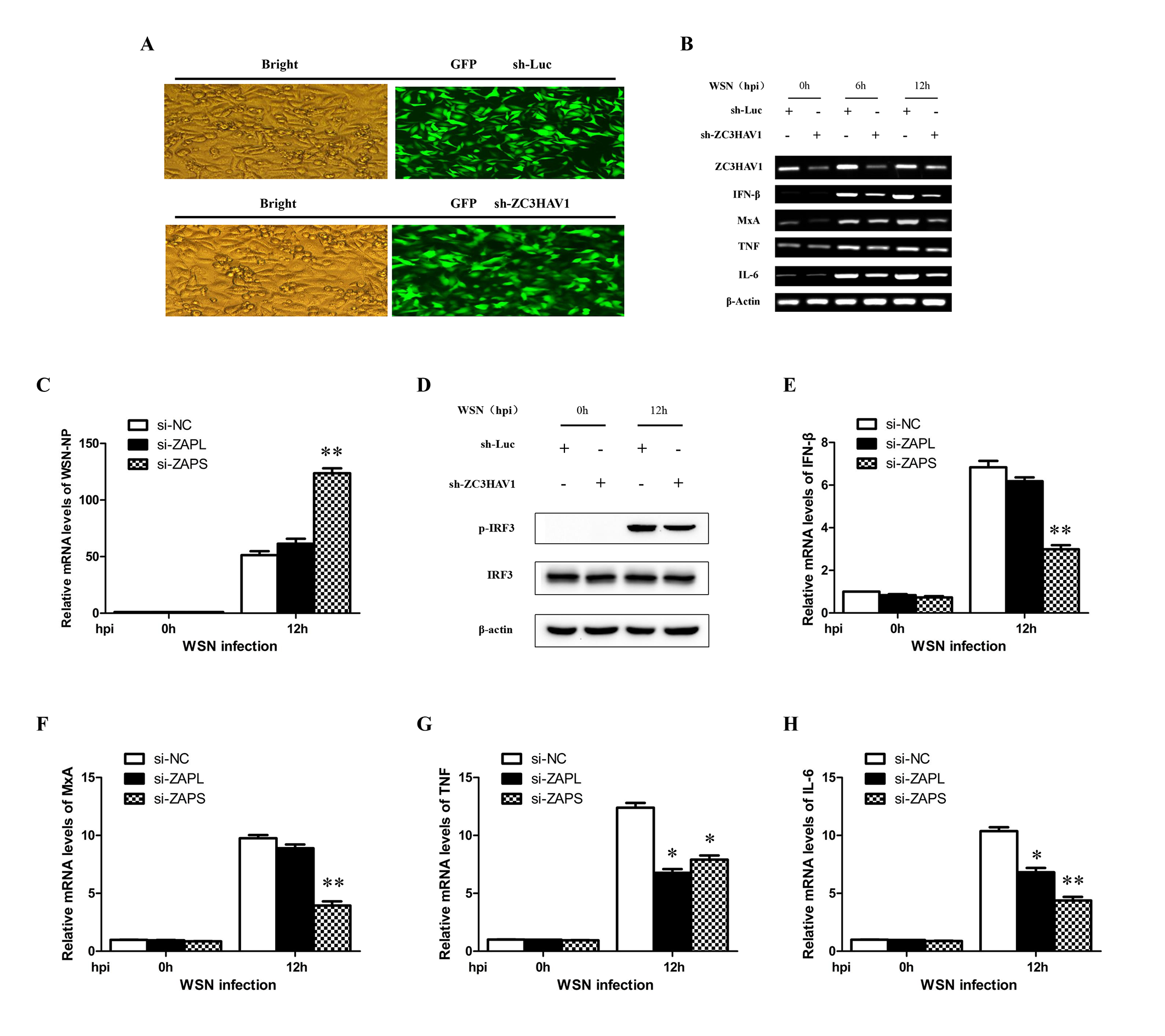

Supplement: FIGURE S1 — (A) The positive cells were observed under fluorescent microscope to detect the expression of GFPs. (B) RT-PCR was performed to detect the expression of ZC3HAV1, IFN-β, MxA, TNF, and IL-6 mRNAs in A549 cells expressing sh-ZC3HAV1 or sh-Luc in response to WSN infection. (D) Western blotting was performed to detect the protein expression of IRF3 and p-IRF3 in ZC3HAV1 knockdown cells after the viral infection. (C,E–H) qRT-PCR was performed to examine the mRNA levels of viral NP, IFN-β, MxA, TNF, and IL-6 in A549 cells expressing either si-NC, si-ZAPL, or si-ZAPS after the WSN infection. Data are represented as mean ± SD. *p < 0.05; **p < 0.01. [file Image_1.tif]

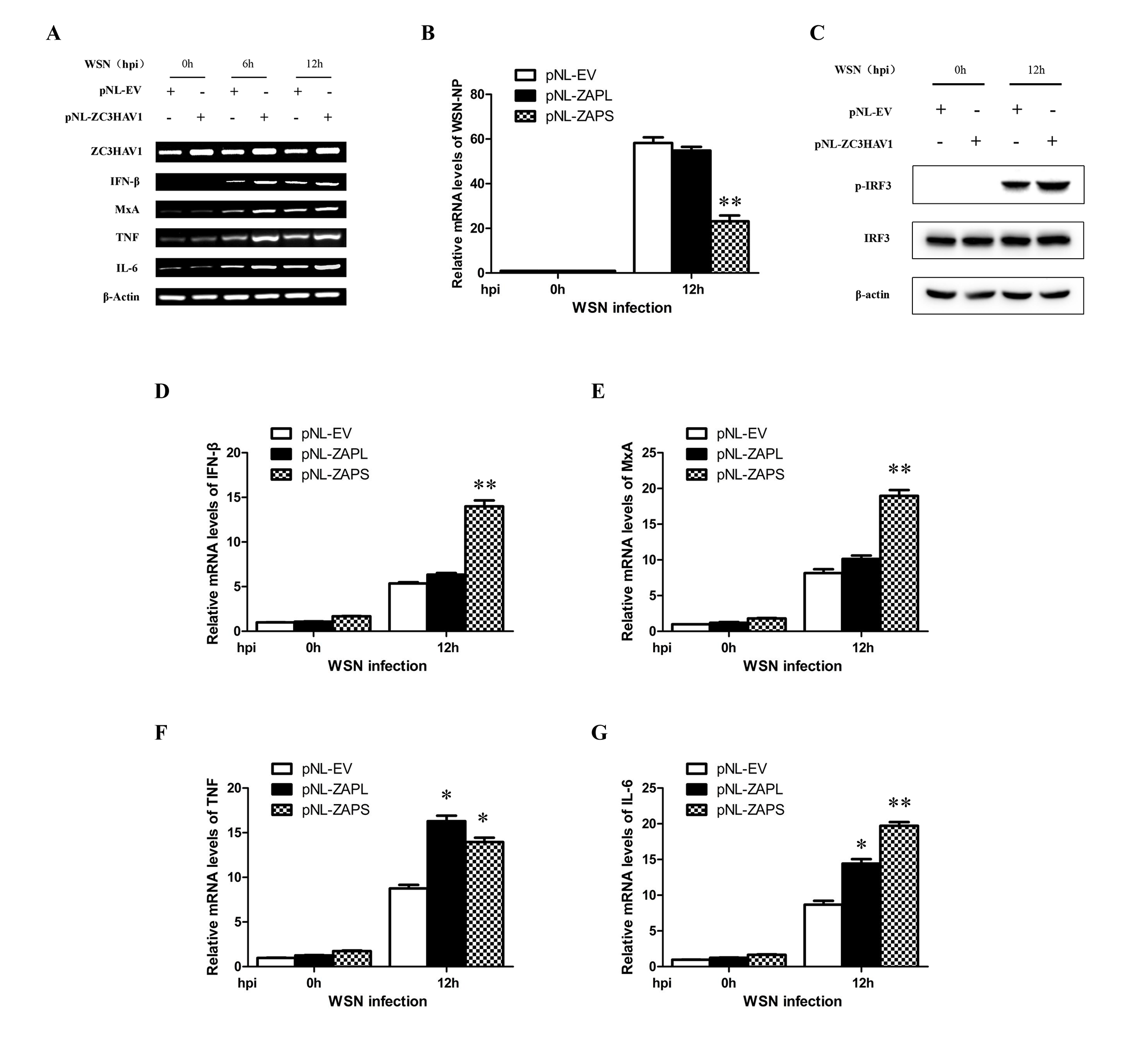

Supplement: FIGURE S2 — (A) RT-PCR was performed to detect the expression of ZC3HAV1, IFN-β, MxA, TNF, and IL-6 mRNAs in A549 cells expressing pNL-ZC3HAV1 or pNL-EV in response to WSN infection. (C) Western blotting was performed to detect the protein expression of IRF3 and p-IRF3 in ZC3HAV1-overexpressed cells after the viral infection. (B,D–G) qRT-PCR was performed to examine the mRNA expression of viral NP, IFN-β, MxA, TNF, and IL-6 in A549 cells overexpressing either pNL-EV, pNL-ZAPL, or pNL-ZAPS. Data are represented as mean ± SD. *p < 0.05; **p < 0.01. [file Image_2.tif]
